# Knowledge, attitudes, and practices regarding tetanus: a case of healthcare workers in the emergency and intensive care departments of a regional hospital in the northern region of Morocco

**DOI:** 10.1186/s12913-025-13522-x

**Published:** 2025-10-08

**Authors:** Nadira Mourabit, Khadija Elwardi, Younes Mahrach, Ouafae Kaissi, Yousra Elboussaadni, Abdallah Oulmaati

**Affiliations:** 1 Laboratory of Research and Development in Engineering Sciences, Faculty of Sciences and Techniques of Al-Hoceima, Abdelmalek EssaâdiUniversity, Tetouan, Morocco; 2 Research Team in Biotechnology and Biomolecule Engineering (ERBGB), Faculty of Science and Technology of Tangier, Abdelmalek Essaâdi University, Tetouan, Morocco; 3 Department of Medical Genetics and Oncogenetics, Centre Hospitalier Universitaire Mohammed 6, Tangier, Morocco; 4 Pediatric Department, Centre Hospitalier Universitaire, Faculty of Medicine and Pharmacy of Tangier, Abdelmalek Essaâdi University, Tetouan, Morocco

**Keywords:** Tetanus, Healthcare workers, Knowledge, Attitudes, Practices, Prevention

## Abstract

**Background:**

Tetanus is a potentially fatal, vaccine-preventable infection caused by *Clostridium tetani*. Healthcare workers play a crucial role in preventing this disease and managing infections in the community.

**Methods:**

A cross-sectional study was conducted between March and August 2023 in the emergency and intensive care units of a regional hospital in northern Morocco to evaluate healthcare workers’ knowledge, diagnostic attitudes, treatment practices, and preventive awareness regarding tetanus. A purposive sampling strategy was used to recruit participants, and data were collected through a structured, self-administered, and anonymous questionnaire. Descriptive statistics summarized demographic characteristics and competencies related to tetanus management. Associations between variables were explored using chi-squared or Fisher’s exact tests, as appropriate, followed by multivariate logistic regression to identify independent socio-demographic predictors. Statistical significance was set at *p* < 0.05, and the strength of associations was expressed as odds ratios (ORs) with corresponding 95% confidence intervals (CIs).

**Results:**

The study included 83 healthcare workers, comprising 72.29% nurses and 27.71% physicians. Among these participants, 62.65% demonstrated limited knowledge about tetanus. The accuracy of disease diagnosis was reported at 43.48% for physicians and 25% for nurses. A significant lack of adherence to treatment guidelines and protocols was noted in 76.66% of nurses and 78.26% of physicians. Furthermore, 60.24% of participants reported either a lack of ongoing training in tetanus management or a lack of access to published guidelines to support their continuous learning. A low level of diagnostic accuracy among healthcare workers was significantly associated with inadequate treatment expertise (OR = 3.63; 95% CI: 1.24–10.58; *p* = 0.02). Additionally, nurses were more likely than physicians to demonstrate low prevention awareness (OR = 3.59; 95% CI: 1.12–12.9; *p* = 0.04).

**Conclusion:**

Our findings indicate substantial gaps in the knowledge and clinical management of tetanus among healthcare workers in the emergency and intensive care units of this hospital. We recommend implementing structured initial and ongoing training programs for both physicians and nurses to enhance the management of tetanus.

**Supplementary Information:**

The online version contains supplementary material available at 10.1186/s12913-025-13522-x.

**Table:** 

Text box 1. Contributions to the literature
• This study examined healthcare workers’ knowledge, attitudes, and practices regarding Tetanus.
• It emphasises the importance of vaccination programs in preventing Tetanus and maintaining healthcare standards in Morocco.
• The study reminds healthcare professionals and policymakers of the importance of vigilance and ongoing efforts in tetanus prevention.

## Background

Tetanus is a potentially fatal, vaccine-preventable, and reportable disease caused by Clostridium tetani, a bacterium commonly found in soil and feces [1]. When the spores of this bacterium enter a wound, they can germinate and release a neurotoxin called tetanospasmin. This toxin disrupts the signaling between nerves and muscles, resulting in muscle stiffness and spasms, particularly in the jaw [1]. This can impair the ability to swallow and breathe, potentially leading to death in severe cases [1, 2]. Diagnosis is primarily clinical and requires urgent hospitalization, with management including toxin neutralization using human tetanus immune globulin or equine antitoxin, wound care, antibiotics, and appropriate immunization depending on the patient’s vaccination history [3]. Vaccination serves as the fundamental strategy for the prevention of tetanus. The World Health Organization (WHO) recommends a primary series of tetanus-containing vaccines starting in infancy, with booster doses every ten years to maintain immunity [1–3]. Global efforts have led to substantial declines in tetanus mortality between 1990 and 2015—90% for neonatal tetanus and 81% for non-neonatal tetanus [4]. However, challenges persist in several countries, particularly those with low vaccination rates or limited access to healthcare [4]. Morocco has prioritized tetanus control since the launch of its Expanded Programme on Immunization in 1981, which evolved into a national program providing free tetanus immunization in all public health facilities through national vaccination days, mobile team visits to remote areas, and systematic vaccinations at primary healthcare centers [5–9]. The childhood schedule includes diphtheria, tetanus, and pertussis (DTP) vaccine at 2, 4, and 6 months, with boosters at 15–18 months and 4–6 years. Adults receive adsorbed tetanus–diphtheria (Td) boosters every ten years, which can safely replace the monovalent tetanus toxoid (TT), including during pregnancy [10–12]. Morocco achieved the WHO’s target of reducing neonatal tetanus to less than one case per 1,000 live births in all districts by 1995 and sustained this status in 2002 [13]. As of 2023, national coverage with tetanus-containing vaccines reached 99% [14]. Only two neonatal and two non-neonatal tetanus cases were reported nationwide in 2021, with none in northern Morocco [15]. As a result, tetanus has increasingly become a neglected condition, receiving limited attention in clinical discussions and training programs. However, despite these achievements, sustaining high coverage—especially for booster doses—remains a challenge. Waning immunity, missed booster opportunities, and rising vaccine hesitancy, intensified during the COVID-19 pandemic, threaten recent progress [16–20]. For example, provincial data from Tangier show women’s tetanus vaccination rates declining from 98% in late 2019 to 60% by the end of 2023. While vaccination is essential, WHO also underscores the importance of hand hygiene, infection control, and proper wound management to reduce tetanus risk in healthcare settings [3, 21]. Healthcare workers play a pivotal role in preventing tetanus through vaccination, education, wound care, and case reporting [21, 22]. A lack of awareness about vaccination, together with insufficient knowledge and skills in diagnosis and treatment, may undermine public confidence in immunization, fuel anti-vaccination sentiment, and pose a serious public health risk [21–23]. This study aims to evaluate the knowledge, diagnostic attitudes, treatment practices, and preventive awareness of healthcare workers in the emergency and intensive care units of a regional hospital in northern Morocco, to identify gaps and guide targeted interventions.

## Methods

### Research design and setting

A cross-sectional, monocentric study was conducted over six months, from March to August 2023, among healthcare workers at the regional hospital in the Tangier-Tétouan-Al Hoceïma region of northern Morocco. The hospital serves a catchment area of over two million people and sees approximately 400 to 450 patients each day. Critically ill patients are admitted to the intensive care unit, while the emergency department provides initial care for acute conditions. During the study period, this regional hospital served as the primary referral center for the area, as there were no university hospitals available and secondary care facilities were limited. As a result, this hospital was the only facility offering emergency services. The hospital operates on a 12/36-hour shift system, with a 36-hour rest following a 12-hour shift.

### Target population

The inclusion criteria focused on frontline healthcare workers who provided initial care for patients with wounds in the emergency department and intensive care unit during the early stages of hospitalization. This group comprised 42 permanent staff members: 27 from the emergency department (including 20 nurses and 7 physicians) and 15 from the intensive care unit (consisting of 11 nurses and 4 physicians). Additionally, there were 50 non-permanent personnel, which included contractual and temporary staff, rotating residents, and interns who were active during the six-month study period. In total, 92 individuals were initially assessed for participation.

### Data collection and questionnaire

A structured, self-administered, anonymous questionnaire was distributed to healthcare workers in both paper and digital formats (via Google Forms). This survey was specifically designed for this study based on a literature review and expert consultations in the fields of epidemiology, public health, vaccination, infectious diseases, and nursing. The questionnaire evaluated general knowledge, attitudes, practices, and prevention awareness about tetanus, ensuring that the content was consistent with WHO guidelines and standard clinical training. Experts evaluated the questionnaire for validity, clarity, and relevance, prompting modifications based on their suggestions. A pilot study was conducted with 10% of the target population to test and refine our survey. The final study included 83 participants. The questionnaire addressed demographic characteristics (gender, age group, professional profile, seniority, and department) and consisted of 13 items divided into four domains: 6 items focused on disease knowledge, 4 on diagnostic attitudes, 2 on treatment practices, and 1 on prevention awareness (Additional file 2).

### Scoring

The knowledge level about the disease was evaluated using 6 questions, with each correct answer worth one point and each incorrect answer worth zero points. A total of 6 points could be obtained. The two categories used to describe the general level of knowledge were low (0–4 points) and high ( > = 5 points).

Four statements assessed diagnostic attitudes toward tetanus infections. These statements were scored 1 (for the correct answer) and 0 (for the incorrect answer). In total, we obtained a range of 0 to 4 points. The two categories used to describe the general level of diagnostic attitudes were low (0–2 points) and high (3 points or more).

Two statements were used to assess treatment practices for infection control of Tetanus. Each statement was evaluated using a two-point scale (1 correct and 0 incorrect). To determine the treatment level, a range of 0 to 2 points was used, with low (< 1 point) and high ( > = 1 point) categories.

One question about continuous training and the availability of readily published guidelines was used to evaluate the prevention awareness of healthcare workers about Tetanus. Each positive response will receive one point and be classified as a high prevention level; the inverse is correct.

### Statistical analysis

All statistical analyses were conducted using R software, version 4.3.2. Categorical variables were presented as frequencies and percentages. To examine various associations, bivariate analyses were performed using either the chi-squared test or Fisher’s exact test, as appropriate. To identify independent predictors of lower levels of knowledge, diagnostic attitudes, treatment practices, and preventive awareness, we carried out multivariate logistic regression analyses. A significance threshold of 5% was set, and the strength of the associations was evaluated by calculating the odds ratio (OR) along with its 95% confidence interval (CI).

## Results

### General characteristics of the study respondents

A total of 83 healthcare workers participated in the study, with a majority being female (54.22%). Most participants (85.54%) were aged between 20 and 29 years. Nurses represented the largest group, accounting for 72.29% of the participants. Additionally, 46.99% of the participants had less than 5 years of work experience, while 53.01% had more than 5 years of experience. Furthermore, 59.04% of those surveyed worked in the emergency unit (Supplementary Table 1, Additional file 1).

### Knowledge level regarding tetanus

The findings show that 62.65% of participants possess a low level of knowledge regarding tetanus (Table 1). Although over 86% of respondents accurately recognised tetanus as a severe, fatal, and reportable disease, only 39.76% understood it as a non-contagious and non-immunising toxic infection. Additionally, 63.86% of participants appropriately associated tetanus with anaerobic bacteria, and 66.27% identified the contamination of a necrotic wound or injury as the mode of transmission for the disease (Table 1).

**Table 1:** Evaluation of the general knowledge about tetanus

Questions	*N*(%)
**How can we define Tetanus disease?**	
Correct: Tetanus is a non-contagious and non-immunizing toxic infection.	33 (39.76%)
Other responses.	50 (60.24%)
**Are the disease case-fatality rates high even where intensive care is available?**	
Correct: Yes, tetanus is a severe and fatal disease.	79 (95.18%)
Other responses.	4 (4.82%)
**Are tetanus cases reportable?**	
Correct: Tetanus is a mandatory reportable disease.	72 (86.75%)
Other responses.	11 (13.25%)
**What is the cause of tetanus?**	
Correct: Tetanus is due to anaerobic bacteria	53 (63.86%)
Other responses.	30 (36.14%)
**What is the primary target of the tetanus toxin upon initial exposure?**	
Correct: The initial target of the tetanus toxin is the nervous system.	43 (51.81%)
Other responses.	40 (48.19%)
**How is the disease transmitted?**	
Correct: Disease transmission occurs through the contamination of necrotic wounds and injuries.	55 (66.27%)
Other responses.	28 (33.73%)
**Overall level of knowledge**	
High ( > = 5 points)	31 (37.35%)
Low (0–4 points)	52 (62.65%)
**Knowledge level**	**Low**

### Diagnostic attitudes toward tetanus

Among the 60 nurses and 23 physicians included in this survey, the accuracy of disease diagnosis was recorded at 43.48% for physicians and 25% for nurses. 61.67% of physicians and 60.87% of nurses were able to correctly identify the signs of adult tetanus as outlined by the WHO. Meanwhile, 73.33% of nurses and 30.43% of physicians who participated in the survey did not recognise the signs associated with confirmed neonatal tetanus. Furthermore, when it comes to understanding the risk factors that lead to the germination of tetanus toxins in injuries, only 20% of nurses and just 13.04% of physicians could identify these crucial elements (Table 2).

**Table 2:** Assessment of the diagnostic attitudes of healthcare workers regarding tetanus

Questions	*N* (%)	
	Nurses	Physicians
**What signs are required by the World Health Organization’s definition of adult tetanus?**		
Correct: trismus or risus sardonicus, or painful muscular contractions.	37 (61.67%)	14 (60.87%)
Other responses.	23 (38.33%)	9 (39.13%)
**How does the World Health Organization define a confirmed neonatal tetanus case?**		
Correct: A newborn with normal sucking and crying ability in the first two days, but loses it and becomes rigid/spastic between days 3–28.	16 (26.66%)	16 (69.57%)
Other responses.	44 (73.33%)	7 (30.43%)
**On what basis is the diagnosis of tetanus established?**		
Correct: The diagnosis of tetanus is based on clinical and interrogation.	31 (51.67%)	17 (73.91%)
Other responses.	29 (48.33%)	6 (26.09%)
**What factors contribute to an elevated risk of toxin germination in wounds?**		
Correct: Deep, dirty wounds in unvaccinated individuals, especially in areas with high soil contamination and poor hygiene/sanitation, with delayed wound care, increase tetanus risk.	12 (20%)	3 (13.04%)
Other responses.	48 (80%)	20 (86.96%)
**The overall level of diagnostic**		
High ( > = 3 points)	15 (25%)	10 (43.48%)
Low (0–2 points)	45 (75%)	13 (56.52%)
**Diagnostic level**	**Low**	

### Treatment practices for tetanus cases

The survey results revealed that 81.67% of nurses and 86.96% of physicians among the participants were unable to accurately identify the primary focus of curative treatment for tetanus, and 95% of nurses and 91.30% of physicians incorrectly identified the tetanus vaccination protocol for post-exposure prophylaxis for both immunized and non-immunized patients (Supplementary Table 2, Additional file 1).

### Preventive awareness of tetanus

60.24% of healthcare workers reported a lack of published guidelines or ongoing training on tetanus prevention measures and vaccination provided by the hospital, indicating a low overall level of preventive awareness among healthcare workers (Supplementary Table 3, Additional file 1).

### Factors associated with healthcare workers’ knowledge, diagnostic attitudes, treatment practices and prevention awareness regarding tetanus management

No statistically significant associations were identified among demographic variables—including gender, age group, professional profile, seniority, and service of attachment—and knowledge levels regarding tetanus, diagnostic attitudes, treatment practices, and awareness of preventive measures (Supplementary Table 4, Additional file 1).

### Impact of knowledge levels on healthcare workers’ Diagnosis, Treatment, and prevention levels of tetanus

The evaluation of the impact of knowledge levels on diagnostic attitudes, therapeutic practices, and preventive awareness related to tetanus reveals no statistically significant associations. In conclusion, individuals with elevated knowledge levels do not demonstrate a substantial difference in their diagnostic attitudes, treatment practices, or awareness of prevention strategies for tetanus compared to those with lower knowledge levels (Supplementary Table 5, Additional file 1).

### Impact of diagnostic attitude levels on healthcare workers’ treatment and prevention levels of tetanus

Participants exhibiting lower diagnostic attitudes are significantly more prone to exhibit inadequate treatment practices, as indicated by a p-value of 0.02 and an odds ratio of 3.63 (95% CI: 1.24–10.58). Conversely, no statistically significant correlation was observed between the levels of diagnostic attitudes and the degree of awareness of disease prevention (Supplementary Table 6, Additional File 1).

### Multivariate analysis

Logistic regression analysis revealed that the only significant predictor of prevention awareness was the professional profile. Nurses were more likely than physicians to demonstrate high prevention awareness (OR = 3.59; 95% CI: 1.12–12.90; *p* = 0.04). No significant associations were observed for gender, age group, seniority, or service. All four models demonstrated good fit (Hosmer–Lemeshow test, *p* > 0.05). Nagelkerke pseudo R² values ranged from 0.02 to 0.13, indicating low explanatory power. No evidence of multicollinearity was detected (all VIF < 2) (Table 3).

**Table 3:** Logistic regression analyses of factors associated with low levels of knowledge, diagnostic Attitudes, treatment Practices, and prevention awareness

Variables	Category	Low level of knowledge		Low level of diagnostic attitudes		Low level of Treatment practices		Low level of Prevention awareness	
		OR(95%CI)	p-value	OR(95%CI)	p-value	OR(95%CI)	p-value	OR(95%CI)	p-value
Gender	Male	0.78(0.31–1.98)	0.61	1.07(0.40–2.90)	0.89	0.98(0.32–2.96)	0.97	0.92(0.35–2.37)	0.86
	Female	1	-	1	-	1	-	1	-
Group age	B: 30–39	0.43(0.08–1.89)	0.29	1.61(0.36–6.86)	0.52	1.45(0.28–6.87)	0.64	1.41(0.30–6.43)	0.65
	A: − 29 years	1	-	1	-	1	-	1	-
Profile	Nurses	0.77(0.25–2.38)	0.65	0.50(0.16–1.55)	0.23	0.89(0.22–3.77)	0.87	3.59(1.12–12.90)	0.04*
	Physicians	1	-	1	-	1	-	1	-
Seniority	>= 5 years	1.15(0.43–3.17)	0.78	0.77(0.26–2.27)	0.63	2.92(0.88–11.2)	0.09	0.37(0.13–1.01)	0.06
	< 5 years	1	-	1	-	1	-	1	-
Service	Emergency	1.02(0.40–2.61)	0.97	0.93(0.34–2.55)	0.88	0.55(0.18–1.63)	0.28	1.72(0.66–4.62)	0.27
	Intensive Care	1	-	1	-	1	-	1	-

*p < 0.05 is considered statistically significant

## Discussion

Tetanus is one of the diseases monitored through epidemiological surveillance in Morocco. It is a serious condition that requires prompt and immediate treatment [15]. Effective prevention depends on up-to-date vaccination and proper wound care by qualified and well-trained healthcare workers [2, 3]. The current study examines the knowledge and attitudes of emergency and intensive care personnel at a regional hospital in northern Morocco regarding tetanus management. The findings indicate significant deficiencies in both theoretical knowledge and practical application. These gaps underscore the need for targeted interventions to enhance clinical competency and improve patient outcomes.

The lack of knowledge and ongoing training among healthcare workers is significantly associated with suboptimal prophylactic practices, missed vaccination opportunities, and ultimately, poor patient outcomes [21–24]. Studies report frequent overuse of tetanus immune globulin (TIG) and underuse of the tetanus vaccine, reflecting deviations from clinical guidelines [22–24]. These gaps can erode public confidence in health initiatives, exacerbate vaccine hesitancy, and impede the efficacy of disease prevention strategies [25, 26]. Conversely, facilities with well-trained staff and clear protocols show improved outcomes, highlighting the necessity of regular training and adherence to guidelines [27, 28].

Beyond foundational knowledge, the ability of emergency and intensive care staff to recognize and diagnose both neonatal and non-neonatal tetanus is essential for timely intervention, particularly in settings with limited diagnostic resources [29, 30]. Notably, Many instances of non-neonatal tetanus arise in unvaccinated individuals, frequently after minor wounds that might not receive medical attention [31–33]. Neonatal tetanus continues to be a significant issue, often associated with insufficient maternal immunisation and inadequate wound care during delivery [31]. Deficiencies in diagnostic skills have been linked to delays in initiating prophylaxis and treatment, increasing symptom severity and mortality risk, given the rapid progression of tetanus [34, 35].

Our research reveals a significant deficiency in the availability and accessibility of tetanus prevention guidelines and training within hospital settings. Notably, nurses have reported this shortcoming more frequently than physicians. This issue is particularly critical in the context of public health in Morocco, where nurses are primarily responsible for administering vaccinations in primary healthcare centers. The implications of this gap could negatively impact patient education and awareness, as those administering the vaccines may lack the necessary expertise. Moreover, the high turnover rate among medical staff presents a significant challenge to delivering comprehensive in-service training. This issue is further compounded by a heavy patient load—averaging 400 to 450 consultations daily—and the need to maintain strict infection control protocols. These pressures are especially intense in a regional hospital that serves as the primary referral center in an area without a university hospital and with limited access to secondary care during the study period. Together with demanding work schedules, these factors contribute to substantial fatigue and stress among healthcare workers. A U.S. study involving 2,310 healthcare workers identified work-related stress (49%), personal stress (32%), and overlapping stressors (19%) as prominent challenges. This underscores the urgent need for implementing stress management strategies and fostering supportive work environments to improve adherence to infection prevention protocols [36].

Finally, the current study highlights the need for further research to investigate the specific factors contributing to the observed gaps in knowledge, diagnostic attitudes, treatment practices, and preventive awareness among these healthcare workers. This information could inform the development of targeted interventions and strategies to improve infection control practices in the hospital.

### Limitations

This study has several limitations that should be acknowledged. First, the relatively short duration of data collection limited the scope of participant inclusion. Additionally, the study was conducted in a single regional hospital in northern Morocco, which may affect the generalizability of our findings to other healthcare settings or regions. The research focused exclusively on emergency and intensive care units, leaving out other departments, such as pediatrics, where cases of neonatal tetanus are more commonly encountered. Furthermore, this was a preliminary investigation that utilized a broad and general survey instrument, which restricted the depth of our analysis. While the exploratory nature of the study was appropriate for an initial assessment, future research should employ more specialized tools and include a wider range of services to achieve a more comprehensive understanding of knowledge and practices related to tetanus management.

## Conclusion

The study’s findings demonstrate substantial deficiencies in both the knowledge and clinical management of tetanus among healthcare workers within the emergency and intensive care units of this hospital. To address these gaps, we strongly recommend implementing structured and mandatory continuous medical education programs focused on tetanus management, including its diagnosis, treatment protocols, and prevention strategies. Furthermore, strategies to address healthcare worker workload and burnout, particularly for less experienced staff, could enhance adherence to protocols and promote a culture of continuous learning and vigilance.

## Supplementary Information

Below is the link to the electronic supplementary material.

Supplementary Material 1

Supplementary Material 2

## Data Availability

The datasets used and analyzed during the current study are provided within additional files 1 and 2.

## Abbreviations

DTP: Diphtheria, tetanus and pertussis
Td: Tetanus and diphtheria
TT: Tetanus toxoid
