# Supplementary material for: Knowledge, attitudes, and practices regarding tetanus: a case of healthcare workers in the emergency and intensive care departments of a regional hospital in the northern region of Morocco
Source: BMC Health Serv Res. 2025 Oct 8;25:1325. doi: 10.1186/s12913-025-13522-x (PMC12506326; doi:10.1186/s12913-025-13522-x)
Supplement: Supplementary file 1 — Supplementary Material 1 [file 12913_2025_13522_MOESM1_ESM.docx]

**Title:** Knowledge, attitudes, and practices regarding Tetanus: a case of healthcare workers in the emergency and intensive care departments.

**Consent Statement**

Participation and Data Use Consent

We appreciate your agreeing to participate in this investigation. Your participation is entirely voluntary, and you can withdraw at any moment without consequences. you consent to the collection, use, and storage of your responses for research purposes as described below:

**Purpose:** The data collected through this questionnaire will be used solely to assess healthcare workers' knowledge, attitudes, and practices regarding Tetanus management in the emergency and intensive care departments of the regional hospital in northern Morocco.

**Confidentiality:** All information you provide will be kept strictly confidential. Your responses will be anonymized; no personally identifiable information will be shared or published.

**Data Storage:** The data will be stored securely and will only be accessible to the research team involved in this study. It will be retained for five years for further analysis and follow-up studies.

**Voluntary Participation:** your participation is entirely voluntary, and you are not obligated to respond to any questions you would instead not answer. Before completing the questionnaire, you can withdraw from the study by either closing the browser window (if you're participating online) or choosing not to return the paper version.

**Contact Information:** If you have any questions about the study or participation, please get in touch with Dr. Nadira Mourabit at [nmourabit@uae.ac.ma](mailto:nmourabit@uae.ac.ma).

By proceeding with this questionnaire, you indicate that you have read and understood the information above and consent to participate in this study under the described conditions.

**Section 1: Demographic Information**

| **1.** | **What is your gender?** | Male | Female |  |
| --- | --- | --- | --- | --- |
| **2.** | **Your age group is?** | 20 – 29 years old | 30 – 39 years old | Other |
| **3.** | **What is your profile?** | Nurse | Doctor |  |
| **4.** | **Seniority?** | A: < 5 years | B: 5 – 10 years | C: > 10 years |
| **5.** | **Are you working for?** | Emergency unit | Intensive care unit |  |

**Section 2: General Knowledge Regarding Tetanus**

1. **How can we define the Tetanus disease?**

Contagious toxic infections

Immunizing

Non-contagious toxic infections

Non-immunizing

I Don't Know

1. **Are the disease case-fatality rates high even where intensive care is available?**

Yes, Tetanus is a severe and fatal disease.

No

I don’t know

1. **Are Tetanus cases reportable?**

Yes

No

I don’t know

1. **What is the cause of Tetanus?**

An aerobic bacterium

An anaerobic bacterium

Parasites

I don’t know

1. **What is the primary target of the Tetanus toxin upon initial exposure?**

Nervous and muscular systems

Muscular system

Nervous system

Immune system

Internal organs

I don’t know

1. **How is the disease transmitted?**

Via saliva or body fluids

Contamination of necrotic wounds and injuries

By air

Touching contaminated surfaces

Infected insect bite

I don’t know

**Section 3: Tetanus diagnosis**

| 1. **What signs are required by the World Health Organization definition of adult Tetanus?** | |
| --- | --- |
|  | Trismus |
|  | Fever and diarrhea |
|  | Dyspnea |
|  | I don’t know |
| 1. **How does the World Health Organization define a confirmed neonatal Tetanus case?** | |
|  | A newborn with normal sucking and crying ability in the first two days but loses it and becomes rigid/spastic between days 3-28. |
|  | A newborn who is unable to suck or cry generally in the first two days of life and who develops rigidity or spasms between days 3-28. |
|  | A premature newborn who develops fever and irritability in the first two weeks. |
|  | A newborn who has difficulty breathing and shows signs of jaundice in the first week of life. |
|  | I don’t know |
| 1. **On what basis is the diagnosis of Tetanus established?** | |
|  | Clinical criteria |
|  | interrogation |
|  | Complementary examinations |
|  | I don’t know |
| 1. **What factors contribute to an elevated risk of wound toxin germination?** | |
|  | Deep, dirty wounds in unvaccinated people |
|  | hygiene and sanitation are poor, with delayed wound care |
|  | Contact of a wound with the soil |
|  | I don’t know |

**Section 4: Treatment practices for Tetanus cases**

| 1. **What is the primary focus of the curative treatment for the disease?** | |
| --- | --- |
|  | Clean and remove debris to stop infection, use antitoxin and muscle relaxants, and vaccination. |
|  | Cleaning and debridement of the wound and using antitoxin and muscle relaxants only. |
|  | Clean and remove debris, use of antitoxin and muscle relaxants, vaccination, and mandatory reporting. |
|  | I don’t know. |
| 1. **What is the Tetanus vaccination protocol for post-exposure prophylaxis?** | |
|  | For non-immunized patients, a 0.5ml Tetanus toxoid injection is needed for any injury or the time since the last vaccination, with an additional 250 IU HTIG injection and antibiotic therapy for major wounds or delayed debridement. |
|  | Completely immunized patients require the same Tetanus toxoid injection for significant wounds, with antibiotic therapy if the last booster was within ten years. If more than ten years have passed since the last booster, an IgTH 250 IU injection is also recommended. |
|  | For non-immunized patients, a 0.5 ml injection of Tetanus toxoid and antibiotic therapy is required, regardless of the type of injury or the time since the last vaccination. |
|  | For completely immunized patients, only 0.5 ml of Tetanus toxoid is required. |
|  | I don’t know |

**Section 5: Prevention measures**

1. **Are readily published guidelines or continuous training available in the hospital regarding Tetanus prevention measures and vaccination?**

Yes

No

I don’t know
