# Supplementary material for: Knowledge, attitudes, and practices regarding tetanus: a case of healthcare workers in the emergency and intensive care departments of a regional hospital in the northern region of Morocco
Source: BMC Health Serv Res. 2025 Oct 8;25:1325. doi: 10.1186/s12913-025-13522-x (PMC12506326; doi:10.1186/s12913-025-13522-x)
Supplement: Supplementary file 2 — Supplementary Material 2 [file 12913_2025_13522_MOESM2_ESM.docx]

**Knowledge, attitudes, and practices regarding tetanus: a case of healthcare workers in the emergency and intensive care departments of a regional hospital in the northern region of Morocco**

**Statistical Analysis**

**Table 1:** Social demographic characteristics of the participants (n=83).

| Variable | N(%) |
| --- | --- |
| Gender | |
| Male | 38 (45.78 %) |
| Female | 45 (54.22%) |
| Group age | |
| A: 20-29 years | 71 (85.54%) |
| B: 30-39 years | 12 (14.46 %) |
| Profile | |
| Nurse | 60 (72.29%) |
| Physician | 23 ( 27.71%) |
| Seniority | |
| A: < 5 years | 39(46.99%) |
| B: >= 5 years | 44 (53.01 %) |
| Service | |
| Intensive Care Unit | 34( 40.96%) |
| Emergency Unit | 49 ( 59.04%) |
| Total | 83 |

**Table 2:** Adherence to treatment guidelines of healthcare workers regarding Tetanus cases.

| **Questions** | **N (%)** | |
| --- | --- | --- |
|  | **Nurses** | **Physicians** |
|  |  |  |
| **What is the primary focus of the curative treatment for the disease?** | | |
| **Correct:** Clean and remove debris to stop infection; Antitoxin neutralizes the toxin to prevent further damage. Muscle relaxants to ease spasms and improve breathing. Vaccination (prevention) is the Key to preventing future infections. Reporting is often mandatory for public health tracking. | 11 (18.33%) | 3 (13.04%) |
| **Other responses:** Cleaning and debridement of the wound, as well as the use of antitoxin only. | 49 (81.67%) | 20 (86.96%) |
| **What is the tetanus vaccination protocol for post-exposure prophylaxis?** | | |
| **Correct:** For non-immunized patients, a 0.5ml injection of tetanus toxoid is required, regardless of the type of injury or the time since the last vaccination. If the wound is significant or if debridement is delayed, an injection of 250 IU of human tetanus immunoglobulin (HTIG) is also recommended, along with antibiotic therapy.  For completely immunised patients, 0.5 mL of tetanus toxoid is required for the primary wound, and antibiotic therapy is recommended for delayed debridement if less than ten years have passed since the last booster. If more than ten years have passed since the last booster, an injection of IgTH 250 IU is also recommended. | 3 (5.00%) | 2 (8.69%) |
| **Other responses:** For non-immunized patients, a 0.5 ml injection of tetanus toxoid only is required, regardless of the type of injury or the time since the last vaccination; for completely immunized patients, only 0.5 ml of tetanus toxoid is needed. | 57 (95.00%) | 21 (91.30%) |
| **The overall level of treatment practices** | | |
| High level (>=1) | 14 (23.33%) | 5 (21.74%) |
| Low level (== 0) | 46 (76.66%) | 18 (78.26%) |
| **Treatment practices level** | **Low** | |

| Question | N (%) | |
| --- | --- | --- |
|  | Yes | No |
| Are readily published guidelines or continuous training available in the hospital regarding tetanus prevention measures and vaccination? | 33(39.76 %) | 50 (60.24%) |
| **Prevention awareness level** | **Low level** | |

**Table 3:** Healthcare Workers’ Awareness of Tetanus Prevention Resources.

**Bivariate Analysis of Factors Influencing Tetanus-Related Knowledge, Attitudes, Practices, and Prevention Awareness Among Healthcare Workers**

**Table 4:** Relationship between demographic characteristics and levels of knowledge, diagnostic attitudes, treatment practices, and prevention awareness regarding tetanus among healthcare workers.

| Characteristics | Knowledge level | | | | Level of diagnostic attitudes | | | | Level of treatment practices | | | | Level of prevention awareness | | | |
| --- | --- | --- | --- | --- | --- | --- | --- | --- | --- | --- | --- | --- | --- | --- | --- | --- |
|  | High (**n=** 31) | Low **(n= 52)** | p-value | OR (95%CI) | High  **(n= 25)** | Low  **(n= 58)** | p-value | OR (95%CI) | High  **(n= 19)** | Low  **(n= 64)** | p-value | OR (95%CI) | High  **(n= 33)** | Low  **(n= 50)** | p-value | OR (95%CI) |
| **Gender** | | | | | | | | | | | | | | | | |
| Female **(n= 45)** | 18 | 27 | 0.75 | 1.28  (0.52 - 3.14 ) | 14 | 31 | 0.99 | 1.11  (0.43 - 2.85) | 10 | 35 | 0.99 | 0.92 (0.33 - 2.57) | 18 | 27 | 0.99 | 0.98  (0.40 - 2.36) |
| Male **(n= 38)** | 13 | 25 |  |  | 11 | 27 |  |  | 9 | 29 |  |  | 15 | 23 |  |  |
| **Group age** | | | | | | | | | | | | | | | | |
| A: 20 - 29 **(n= 71)** | 28 | 43 | 0.52 | 1.95  (0.49 - 7.85) | 20 | 51 | 0.50 | 0.55  (0.16 - 1.93) | 15 | 56 | 0.46 | 0.54 (0.14 - 2.02) | 29 | 42 | 0.76 | 0.72  ( 0.20 - 2.63) |
| B: 30 - 39 **(n= 12)** | 3 | 9 |  |  | 5 | 7 |  |  | 4 | 8 |  |  | 4 | 8 |  |  |
| **Profile** | | | | | | | | | | | | | | | | |
| Nurses **(n= 60)** | 22 | 38 | 0.99 | 0.90  (0.34 - 2.42) | 15 | 45 | 0.17 | 0.43  (0.16 - 1.19) | 14 | 46 | 0.99 | 1.10 ( 0.34 - 3.49) | 27 | 33 | 0.19 | 0.43  ( 0.15 - 1.25) |
| Physicians **(n= 23)** | 9 | 14 |  |  | 10 | 13 |  |  | 5 | 18 |  |  | 6 | 17 |  |  |
| **Seniority** | | | | | | | | | | | | | | | | |
| A: < 5 years **(n= 39)** | 15 | 24 | 0.99 | 1.09  (0.45 - 2.67) | 13 | 26 | 0.72 | 1.33  (0.52 - 3.41) | 5 | 34 | 0.07 | 0.32 (0.10 – 0.98) | 19 | 20 | 0.18 | 0.49  ( 0.20 - 1.20) |
| B: >= 5 years **(n= 44)** | 16 | 28 |  |  | 12 | 32 |  |  | 14 | 30 |  |  | 14 | 30 |  |  |
| **Service** | | | | | | | | | | | | | | | | |
| Intensive Care Unit **(n= 34)** | 13 | 21 | 0.99 | 1.07  ( 0.43 - 2.63) | 10 | 24 | 0.99 | 0.94  (0.36 - 2.46) | 10 | 24 | 0.36 | 1.85 ( 0.66 - 5.20) | 11 | 23 | 0.36 | 1.7  ( 0.68 - 4.24) |
| Emergency **(n= 49)** | 18 | 31 |  |  | 15 | 34 |  |  | 9 | 40 |  |  | 22 | 27 |  |  |

**Table 5:** The influence of knowledge level on diagnostic attitudes, treatment practices, and prevention awareness regarding tetanus among healthcare workers.

| Knowledge level | Level of diagnostic attitudes | | | | Level of treatment practices | | | | Level of prevention awareness | | | |
| --- | --- | --- | --- | --- | --- | --- | --- | --- | --- | --- | --- | --- |
|  | Low | High | p-value | OR (95%CI) | Low | High | p-value | OR (95%CI) | Low | High | p-value | OR (95%CI) |
| Low | 37 | 15 | 0.74 | 1.17 ( 0.45 – 3.08 ) | 42 | 10 | 0.31 | 1.72 (0.61 - 4.85) | 30 | 22 | 0.54 | 0.75 (0.30 – 1.88 ) |
| High | 21 | 10 |  |  | 22 | 9 |  |  | 20 | 11 |  |  |

**Table 6:** The influence of diagnostic attitude levels on treatment practices and prevention awareness regarding tetanus among healthcare workers.

| Level of diagnostic attitudes | Level of treatment practices | | | | Level of prevention awareness | | | |
| --- | --- | --- | --- | --- | --- | --- | --- | --- |
|  | Low | High | p-value | OR (95%CI) | Low | High | p-value | OR (95%CI) |
| Low | 49 | 9 | 0.02* | 3.63 (1.24 – 10.58) | 39 | 19 | 0.08 | 0.38 (0.15 - 1.00 ) |
| High | 15 | 10 |  |  | 11 | 14 |  |  |

*p < 0.05 is considered statistically significant
